# Susceptibility of human primary neuronal cells to Xenotropic Murine Leukemia Virus-related (XMRV) virus infection

**DOI:** 10.1186/1743-422X-8-443

**Published:** 2011-09-20

**Authors:** Veerasamy Ravichandran, Eugen O Major, Carol Ibe, Maria Chiara Monaco, Mohan Kumar Haleyur Girisetty, Indira K Hewlett

**Affiliations:** 1 Laboratory of Molecular Virology, Division of Emerging and Transfusion Transmitted Diseases, Office of Blood Research and Review, Center for Biologics Evaluation and Research, Food and Drug Administration, Bethesda, MD 20892, USA; 2 Laboratory of Molecular Medicine and Neuroscience, National Institute of Neurological Disorders and Stroke, National Institutes of Health, Bethesda, MD 20892, USA

**Keywords:** XMRV infection, Primary neuronal cells, Non-neuronal cells, Nuclear factors

## Abstract

**Background:**

Xenotropic Murine Leukemia Virus-related (XMRV) virus is a recently identified mouse gammaretrovirus that has the ability to infect certain human cells. In this study, we investigated the susceptibility of primary neuronal cell types to infection with XMRV.

**Findings:**

We observed that the human primary progenitors, progenitor-derived neurons, and progenitor-derived astrocytes supported XMRV multiplication. Interestingly, both progenitors and progenitor-derived neurons were more susceptible compared with progenitor-derived astrocytes. In addition, XMRV-infected Jurkat cells were able to transmit infection to neuronal cells.

**Conclusions:**

These data suggest that neuronal cells are susceptible for XMRV infection.

## Findings

**Figure 1:**
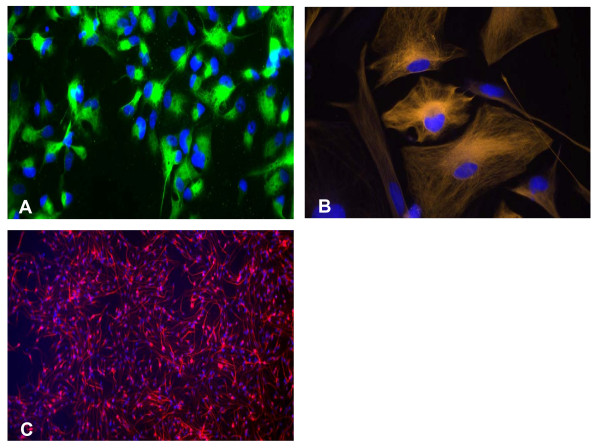
**Human neuronal cells used for the XMRV infection study**: **A**. Human neural progenitor cells (NPCs) generated from a human fetal brain. Progenitor cells were labeled with human nestin-specific goat anti-mouse FITC (green) monoclonal antibody (Invitrogen, A10530). Nuclei were stained with DAPI. **B**. Progenitor-derived Astrocyte (PDA). PDAs express GFAP. Cells were stained using donkey anti-rabbit IgG-TRITC (orange) secondary antibody (Jackson ImmunoRes, 711-025-152). Nuclei are shown in blue (DAPI). **C**. Progenitor-derived neurons (PDN). PDNs express the neuron-specific class III β-tubulin (TUJ1) marker. Cells were stained using goat anti-mouse Alexa Fluor 647 (Red) secondary antibody (Invitrogen: A21241). Nuclei were stained with DAPI (blue).

XMRV has been associated with prostate cancer and CFS [1,2]. Although CFS patients show many symptoms for inflammation in brain, there is no report about the presence of XMRV in the brain of CFS patients. In an effort to determine the susceptibility of neuronal cells to XMRV in-vitro, we used human primary Progenitors, Progenitor-Derived Neurons (PDN), and Progenitor-Derived Astrocytes (PDA). Human central nervous system progenitor cells were isolated from an 8-week-gestation fetal brain according to National Institutes of Health guidelines. The isolation and culture conditions of the progenitor cells and subsequent selective differentiation into neurons were previously reported [3]. Progenitor cultures stained positive for nestin staining (Figure 1A). Differentiation of progenitors into an astrocytic lineage PDA was initiated by growing the cells in Eagle minimal essential medium supplemented with 10% fetal bovine serum (Quality Biologicals, Gaithersburg, MD) (Figure 1B). Progenitors were differentiated into a neuronal phenotype (MAP-2+, βIII-tubulin+, and negative for nestin) by culturing in 10 ng/ml brain-derived neurotrophic factor and 10 ng/ml platelet-derived growth factor A/B (Sigma, St. Louis, Mo.) (Figure 1C).

XMRV (10^8 ^copies/mL) was used to infect progenitors, PDN, and PDA for two hours, and then washed, replenished with appropriate fresh neuronal media. Four days after the infection, cells were washed four times, and harvested for total RNA isolation. Real-time quantitative PCR was performed with Master Mix (Quantitect Probe RT-PCR, Qiagen) using forward and reverse primers encoding gag region of XMRV, probe, RT-mix, and 1 ug of RNA in a total volume of 25 uL. The mixture was incubated at 50°C for 20 min, at 95°C for 10 min, and then cycled at 95°C for 15 sec and 60°C for 60 sec 40 times, using the Applied Biosystems 7500 sequence detection system. XMRV levels were quantitated using the XMRV clone VP62-pcDNA3.1 (GenBank accession no. EF185282; obtained through NIH AIDS Research and Reference Reagent Program) as a standard. Expression levels of XMRV were normalized against glyceraldehyde-3-phosphate dehydrogenase (GAPDH) RNA expression.

**Figure 2:**
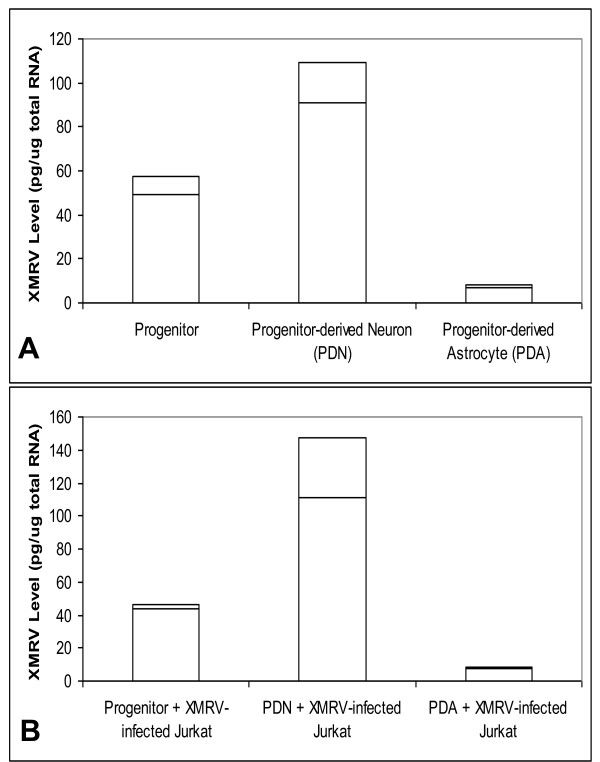
**Infection of XMRV in Primary Neuronal cell types**. **A**. Progenitors, PDNs, or PDAs are plated on 24-well poly d-lysine coated plates (2.5 × 10^5 ^cells/well). XMRV (10^7 ^copies/mL) was exposed these cells for two hours. Four days post-infection, XMRV levels were measured from the total RNA. Results are from three independent experiments. **B**. Infection of XMRV on Neuronal Cells Co-cultured with XMRV-infected Jurkat cells. XMRV-infected Jurkat cells are exposed on the adherent neuronal cells for four hours. The Jurkat cells were washed and the XMRV from the neuronal cells were measured. Results are from three independent experiments.

As shown in Figure 2A., four days post-infection, PDN showed higher susceptibility to XMRV infection, having about 90 pg of XMRV RNA/ug of total cellular RNA. The infected progenitors contained about 50 pg of XMRV RNA/ug of total cellular RNA. Interestingly, infected PDA had levels around 7 pg of XMRV RNA/ug of total cellular RNA. We also measured XMRV levels in the culture media, and found a similar trend (results not shown).

To compare the infectivity of XMRV in neuronal cells with non-neuronal cell types, the average XMRV levels measured four days post-XMRV infection in various cell types are presented in Table 1. Of many cell types tested for the XMRV infectivity, we observed maximal infectivity in LNCaP cells. This is in agreement with reported studies that the human prostate adenocarcinoma cell line LNCaP which provides optimal environment for the XMRV multiplication. Interestingly, the neuronal cells showed high susceptibility to XMRV compared with another human prostate carcinoma cell line DU145.

**Table 1:** Comparison of XMRV infection

Cell	Type	XMRV level (pg/ug total RNA)
Progenitor	Primary neuronal	50.0

Progenitor-derived Neuron	Primary neuronal	90.0

Progenitor-derived Astrocyte	Primary neuronal	7.2

Jurkat	Immortalized T-cell	4.8

DU145	Human prostate carcinoma	3.8

LNCaP	Human prostate adenocarcinoma	516

293T	Human embryonic kidney	0.158

Neuronal and non-neuronal cells were exposed with XMRV (10^7 ^copies/mL) for two hours. Four days post-infection, XMRV levels were measured from the total RNA.

T-cell traffic into the central nervous system is thought to occur when activated T-cells cross the blood-brain barrier [4]. The T-cells presumably act as a "Trojan horse" to store and transport infectious materials across the blood-brain barrier. This "Trojan horse" hypothesis has been well established in the pathogenesis of many viruses that infect the central nervous system [5-7]. Based on the assumption that XMRV infected lymphocytes could infiltrate and infect the neuronal cells, we conducted an in-vitro co-infection/co-culture study using XMRV-infected Jurkat cells with neuronal cells.

To produce XMRV-infected Jurkat cells, Jurkat cells were exposed with XMRV (1.0 × 10^8 ^copies of XMRV/mL) for two hours, washed and replenished with fresh media. Four weeks after the infection, cells were washed four times, and harvested for total RNA isolation. The XMRV levels were measured using Real-time PCR method as described above. The XMRV-infected Jurkat cells had levels around 500 pg of XMRV per ug of total RNA. For the co-infection/co-culture experiment, 1.0 × 10^6 ^XMRV-infected Jurkat cells (XMRV equivalent of 500 pg) were pelleted, treated with trypsin for 3 min at room temperature, washed and suspended in progenitor/PDN/PDA media; and added on to the appropriate adherent neuronal cell types (progenitors, PDN, or PDA) grown in 24 well plate (2.5 × 10^5 ^cells/well). Four hours after co-infection/co-culture, XMRV-infected Jurkat cells were removed, and the adherent neuronal cells were washed with appropriate neuronal media. To remove any adhered Jurkat cells from neuronal cells, each culture well was treated with trypsin, and just before the cells were ready to detach (after about 3 min), fresh neuronal media were added and washed three times using neuronal media. The resulting adherent neuronal cells were free from Jurkat cells, and continued to grow with appropriate neuronal media. After four days, the neuronal cells were harvested for total RNA isolation, and XMRV levels were quantified.

As in the neuronal infection study (Figure 2A), the co-culture/co-infection of XMRV-infected Jurkat (XMRV equivalent of 500 pg) with neuronal cells resulted in proportionate XMRV infection in neuronal cells (Figure 2B). In a separate co-culture/co-infection experiment, the XMRV-infected Jurkats (XMRV equivalent of 4.8 pg) were added with individual neuronal cells (progenitor, PDN, or PDA) as described above, continued for four days. We observed that even the small amount of XMRV in the XMRV-infected Jurkats could release virus and infect the neuronal cells (results not shown). Interestingly, in the co-cultures/co-infection study, we co-culture the XMRV-infected Jurkats with neuronal cells only for four hours. The released virus during this time was able to infect the bystander neuronal cells. These observations support the possibility that the infiltrated XMRV-infected T-cells could infect neuronal cells.

Brain cells are protected from pathogens by the blood-brain barrier. Even though neuronal cells are susceptible for XMRV infection, this virus should first cross the blood-brain barrier to inflict any damage to the brain cells in vivo. After XMRV is transported to neuronal cells, the virus needs favorable multiplication environment for its multiplication. For example, we have observed the requirement of nuclear factor I-A (NF-IA) for the XMRV multiplication in Jurkat cells (unpublished observation). Interestingly, both progenitors and neurons express higher levels of NF-IA [8], and this could account for the optimal XMRV multiplication in these cells. In addition, PDA, which is shown to express low NF-IA level compared to progenitor and PDN [8], showed lower susceptibility to XMRV.

In a recent animal study, XMRV was administered to rhesus macaques through intravenous inoculation [9]. Interestingly, 291 days post-infection, infected cells were detectable by fluorescence in situ hybridization (FISH) in brain, showing the possibility of XMRV infection in the brain. This study further substantiates our speculation that XMRV could infect brain cells. Further in-vitro and in-vivo studies are warranted to identify and characterize the XMRV, and its role if any, in the brain cells.

## Competing interests

There are no potential conflicts of financial or personal conflicts of interest related to the results communicated in this paper.

## Authors' contributions

All authors read and approved the final manuscript. VR, CI, MCM, MKHG performed experiments; VR, IKH contributed to designing research, analyzing data, discussing findings, and writing the manuscript; EOG, IKH supervised the work.
